# Variations in the Phagosomal Environment of Human Neutrophils and Mononuclear Phagocyte Subsets

**DOI:** 10.3389/fimmu.2019.00188

**Published:** 2019-03-01

**Authors:** Juliet R. Foote, Amit A. Patel, Simon Yona, Anthony W. Segal

**Affiliations:** Division of Medicine, University College London, London, United Kingdom

**Keywords:** neutrophil, monocyte, macrophage, dendritic cell, phagocytosis, pH, lysosome, NOX2

## Abstract

The phagosome microenvironment maintains enzyme activity and function. Here we compared the phagosomal pH of human neutrophils, monocytes, dendritic cells (DC), and monocyte-derived cells. An unexpected observation was the striking difference in phagosomal environment between the three monocytes subsets. Classical monocytes and neutrophils exhibited alkaline phagosomes, yet non-classical monocytes had more acidic phagosomes, while intermediate monocytes had a phenotype in-between. We next investigated the differences between primary naïve DC vs. *in vitro* monocyte-derived DC (MoDC) and established that both these cells had acidic phagosomal environments. Across all phagocytes, alkalinization was dependent upon the activity of the NADPH oxidase activity, demonstrated by the absence of NADPH oxidase from a patient with chronic granulomatous disease (CGD) or the use of a pharmacological inhibitor, diphenylene iodonium (DPI). Interestingly, MoDC stimulated with bacterial lipopolysaccharide had increased phagosomal pH. Overall, the increase in alkalinity within the phagosome was associated with increased oxidase activity. These data highlight the heterogeneous nature and potential function of phagocytic vacuoles within the family of mononuclear phagocytes.

## Key Points

- Phagosomal function depends upon the action of enzymes released into them from cytoplasmic granules.
- The substantial differences in the phagosomal pH in the different phagocytes will affect their compliment of enzymes and their functions.

## Introduction

The ability to internalize particles is an evolutionary conserved process. The downstream purpose of this process varies, from a simple feeding mechanism to a fundamental component of host defense. Within the professional phagocyte family of cells, neutrophils, macrophages, and monocyte-derived cells predominantly kill and digest microbes, whereas dendritic cells (DC) are principally involved in antigen presentation. Because all these cells are phagocytic in nature, many of their primary functions are conducted within the phagocytic vacuole, it is therefore critical to understand each cell's phagosomal microenvironment and function.

Previous studies in neutrophils demonstrated that their phagosomal pH is elevated between 8.5 and 9 for at least 30 min following phagocytosis (1, 2). This alkalinization together with the influx of potassium ions (3) activates the neutral proteases released from cytoplasmic granules that kill and digest the ingested pathogen. The alkalinization in neutrophil phagosomes is accomplished by the activity of the NADPH oxidase, NOX2. Superoxide is transported by NOX2 into the phagosome where it forms products such as H_2_O_2_ and H_2_0, consuming protons (1).

This NOX2 electron transport chain is also present in monocytes, macrophages and DC (4, 5). Previous studies have demonstrated that NADPH oxidase elevates the pH of phagosomes in monocyte-derived cells stimulated with LPS and IFN-γ termed “M1/classically activated” (6) and has also been shown to be important for antigen processing by DC (7) –although questions remain as to how this is accomplished (8).

Blood monocytes represent a versatile population of cells, composed of several subsets which differ in morphology and transcriptional profiles, described by their location in the blood (9–13). These monocyte subsets can be distinguished by the membrane expression of CD14 and CD16 in humans (13) into CD14^+^ CD16^−^ (Classical) monocytes, CD14^+^ CD16^+^ (Intermediate), and CD14^lo^ CD16^+^ (Non-classical) monocytes (14–16). Monocytes make up around 10% of circulating white blood cells, of which classical monocytes up ~85% of all monocytes, with intermediate and non-classical monocytes making up the remainder. Classical monocytes are rapidly recruited to sites of infection (17, 18). Non-classical monocytes have been termed patrolling monocytes as they are continuously examining endothelial cell integrity (19).

Dendritic cells bridge the gap between innate and adaptive immunity allowing the immune system to mount precise targeted responses. A key function of the DC is to sense foreign antigen in tissues and present it to T cells, initiating the adaptive immune response (20). DC consist of a heterogeneous family of cells that can be classified into plasmacytoid DC (pDC) or conventional DC (cDC). cDC can be further divided into cDC1 or cDC2 (21). cDC1 cells are known to efficiently prime CD8^+^ T cells via cross presentation (22, 23), while, cDC2 have a broad spectrum of activity and can polarize T cells toward Th1, Th2, and Th17 development depending on the antigen presented (24).

Phagosomal function depends upon the release of the contents of the cytoplasmic granules into the phagosome. These are largely enzymes whose optimal pH will vary widely, and differences in pH are expected to have a major influence upon cellular function.

Accordingly, we have undertaken a study to examine this parameter in the different subtypes of mononuclear phagocytes and neutrophils. In addition, we measured NADPH oxidase activity, as this in turn regulates the pH. Major differences in these parameters in the different cell types examined were observed and are described below.

## Materials and Methods

### Ethics Approval

This patient study was carried out in accordance with the recommendations of the Joint UCL/UCLH Committees on the Ethics of Human Research (Project numbers 02/0324 and 10/H0806/115) with written informed consent from all subjects, also in accordance with the Declaration of Helsinki. The CGD patient has a mutation in the CYBB gene: c.517delC, predicting p.Leu173CysfsX16.

### Experimental Buffer

Balanced salt solution (BSS) buffer contained 156 mM NaCl, 3.0 mM KCl, 1.25 mM KH_2_PO_4_, 2 mM MgSO_4_, 2 mM CaCl_2_, 10 mM glucose, 10 mM Hepes at pH 7.4.

### Flow Cytometry Antibodies

For neutrophil and monocyte isolation purity analysis: (from BioLegend unless otherwise stated) CD3 (FITC, HIT3a); CD19 (FITC, HIB19); CD20 (FITC, 2H7); CD56 (FITC, MEM-188); CD66b (AF700, G10F5); HLA-DR (V500, G46-6, BD Biosciences); CD14 (PE, M5E2); CD16 (APC-Cy7, 3G8).

For isolation of mononuclear subsets by FACS: (from BioLegend unless otherwise stated) CD1c (PE-Cy7, L161); CD3 (FITC, HIT3a); CD11c (V450, B-ly6, BD Biosciences); CD14 (PE, M5E2); CD16 (APC-Cy7, 3G8); CD19 (FITC, HIB19); CD20 (FITC, 2H7); CD56 (FITC, MEM-188); CD66b (AF700, G10F5); CD123 (PerCP-Cy5.5, 7G3); HLA-DR (V500, G46-6 BD Biosciences).

For MoDC differentiation analysis: CD1a (BV510, BD Biosciences); CD1c (BV421, BD Biosciences); CD11c (PE-Cy7, BioLegend); CD14 (BV711, BioLegend); CD16 (PE, BD Biosciences); CD64 (FITC, BD Biosciences); CD141 (APC, Miltenyi Biotec).

For macrophage differentiation analysis: CD80 (APC, clone 2D10); APC isotype control; CD200 receptor (PE, clone OX-108); PE isotype control; CD1a (FITC) FITC isotype control (All from BioLegend).

### Cell Isolation and Cell Culture

Blood was obtained in heparin vacutainers from healthy volunteers. Isolation of neutrophils: Human neutrophils from blood were isolated by dextran sedimentation followed by centrifugation through Lymphoprep™ (Axis Shield), and hypotonic lysis to remove erythrocytes. Lymphoprep™ or Ficoll™ was used as density gradient mediums, neither has been shown to have any differential effect on cell preparations (25).

Isolation of blood mononuclear cells by cell sorting: Monocytes were separated from the interphase layer of whole blood when passed through a density gradient medium (Ficoll™, GE Healthcare), then resuspended in PBS containing 2% FCS and 2 mM EDTA. Cells were incubated with CD3 MicroBeads (Miltenyi) to deplete CD3-postive cells using MACS Cell Separation (Miltenyi). CD3-negative enriched cells were incubated with Human Trustain FcX (BioLegend) before antibody labeling. Cell sorting was performed using FACS Aria II (BD Biosciences) as described previously (26): classical (CD14^+^CD16^−^), intermediate (CD14^+^CD16^+^) and non-classical (CD14^lo^CD16^+^). The DC isolation strategy is described in the legend of **Figure 3** which uses the same antibody cocktail as monocyte subset isolation. cDC2 were identified as being HLA-DR^+^, Lin^−^, CD14^−^ CD16^−^, CD123^−^, CD11c^+^ Cd141^−^, and CD1c^+^.

Generation of polarized monocyte-derived cells: monocyte-derived cells were polarized from monocytes, isolated as described above, by method described by Canton et al. (6), in brief: between 8 and 9 × 10^5^ monocytes/well were cultured in an Ibidi μ-Slide 8 well-plate (Ibidi, Germany) in RPMI 1,640 with 10% fetal bovine serum, 500 U/ml antibiotics (penicillin and streptomycin, ThermoFisher), and 10 mM Hepes buffer (Sigma). For the generation of M1 monocyte-derived cells, the culture medium was supplemented with 60 ng/ml GM-CSF for 5 days, then for a further 2 days with 500 ng/ml LPS, and 60 ng/ml IFN-γ. For M2 monocyte-derived cells, 60 ng/ml M-CSF was added for the first 5 days, then 60 ng/ml IL-4 for the final 2 days.

Generation of monocyte-derived dendritic-like cells: monocytes separated from the interphase layer of whole blood on Ficoll™ were further processed with the Human monocyte enrichment kit (Easy Sep) to isolate classical monocytes. 6 × 10^6^ monocytes were cultured for 7 days in 10 cm dishes with 150 ng/ml GM-CSF and 75 ng/ml IL-4 in complete RPMI medium to generate MoDC (27, 28). For stimulation with LPS (from Salmonella abortus equi S-form (TLRgrade™), Enzo life sciences), after 6 days the dish medium was replaced with complete medium also containing 1 μg/ml LPS for another 24 h (29).

### Flow Cytometry

All cells were resuspended in FACS buffer and incubated with Human Trustain FcX (BioLegend) on ice before antibody labeling to reduce non-specific binding. All experiments were carried on the BD LSRFortessa X20 cell analyzer.

### Phagosomal pH Measurements

We have previously described the measurement of phagosomal pH in detail (30). Briefly, the wells of a microscopy plate (μ-Slide Angiogenesis by Ibidi) were washed twice with BSS buffer to remove non-adherent cells, then buffer containing 1 μg/ μl carboxy SNARF-1, AM ester acetate (ThermoFisher) was added for 25 min to label the cytosol, and then washed off with BSS buffer. The microscopy plate was mounted on a 37°C heated stage with or without 5 μM DPI for 15 min for acclimatization before adding approximately 1 × 10^6^ heat-killed *Candida albicans* (strain ATCC 10231 grown from vitroids, Sigma) labeled with SNARF-1 carboxylic acid acetate succinimidyl ester (ThermoFisher) and opsonised with human serum IgG (Vivaglobin). The cells were monitored using a 63 × oil immersion on a Zeiss 700 confocal microscope. A snapshot was taken once a minute for 30 min when the cells were excited at 555 nm and the emission measured at 560–600 nm and 600–610 nm.

The vacuolar pH was measured using a custom macro within the imaging software ImageJ (31). At least 20 cells were analyzed for each condition within one experiment, *n* = 3 unless otherwise stated. The SNARF fluorescence ratio values were converted to pH using the standard curves as described by Levine et al. (1): the fluorescence ratios of extracellular SNARF-labeled *Candida* were measured in different buffer solutions (100 mM KCl with 50 mM buffer salt) from pH 3–13 to construct two standard curves; the fluorescence ratios of SNARF-labeled *Candida* engulfed by human neutrophils were measured after the phagocytosing cells were then subjected to the same buffers with 0.3% saponin; cytoplasmic pH was measured in human neutrophils in the same buffers with nigericin (32).

### Measurement of Phagocytosis

At the end of the kinetic phagosomal pH experiments, trypan blue was added to the cells to quench extracellular Candida fluorescence. Z stacks (8 or 9 1–2 μm sections) were taken in two different random areas of the well for each condition in each experiment, and the total number of cells and total number of cells with at least one engulfed particle were counted using built-in microscope software (Zen, Zeiss).

### Amplex UltraRed Assay

The assay was carried out as previously described (33) with 50,000 cells in each well of a 96 well-plate (Corning). In brief: 50 IU/ml of horseradish peroxidase (Sigma) and 6 μM Amplex ultrared reagent (ThermoFisher) was added to the medium. A basal reading of 3 cycles was recorded before the cells were stimulated by pump injection with 3 μM PMA (Sigma). The fluorescence produced by the oxidation of the Amplex by hydrogen peroxide was measured 30 s for 50 min at 590 nm after excitation at 540 nm in an Omega FluoStar plate reader (BMG Labtech) with three technical replicates for each condition.

### Statistics

Unless otherwise stated, all experiments were repeated three times with three technical repeats. Each graph shows the mean with standard error. Statistical significance was calculated using one-way ANOVA analyses with Bonferroni's multiple comparison test using the GraphPad Prism version 7 (GraphPad software, La Jolla California, USA).

## Results

**Figure 1:**
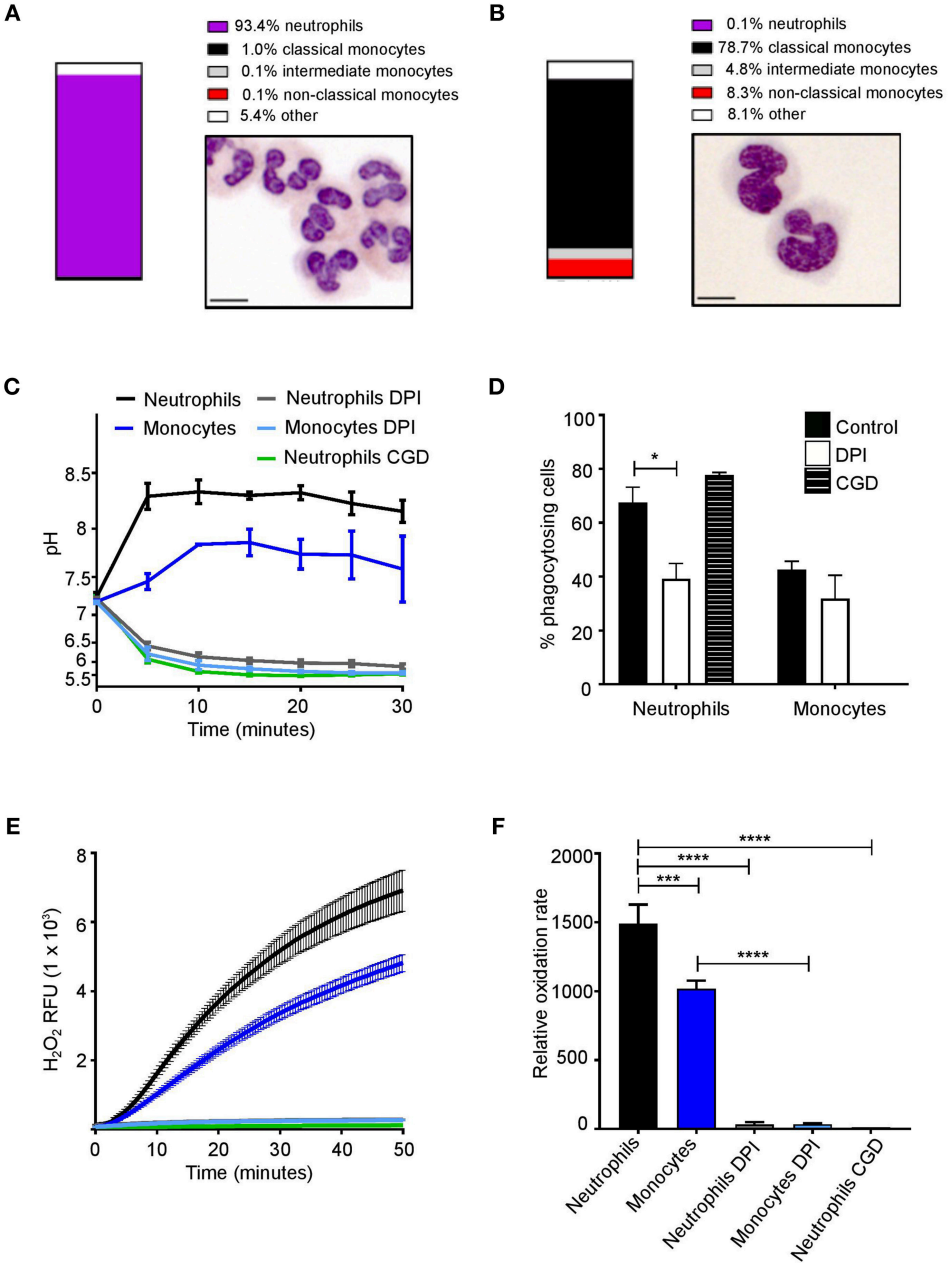
Phagosomal pH and NOX2 activity in human neutrophils and monocytes. Isolation purity and Wright-Giemsa stained preparations of **(A)** neutrophils and **(B)** monocytes. **(C)** Time course of changes in phagosomal pH when cells were challenged with SNARF-labeled Candida, with **(D)** quantitation of the phagocytosis (mean ± SEM). **(E)** Time course of changes in NADPH oxidase activity measured as hydrogen peroxide (H_2_O_2_) induced relative fluorescence units (RFU) after stimulation with PMA, mean (solid lines) ± SEM (dashed lines) and **(F)** maximal respiratory rate. These parameters were tested in cells from a healthy control with and without 5 μM DPI (*n* = 3), an inhibitor of NOX2, and in a patient with X-linked CGD (*n* = 1). Calculated *p*-values from <one-way ANOVA with Bonferroni post-test analysis: ^*^p < 0.05, ^***^p < 0.001, ^****^p < 0.0001. The statistics for **(C)** and **(E)** were: *p* < 0.0001 between neutrophils and DPI or CGD; monocytes and DPI; and between neutrophils and monocytes. No significance between DPI and CGD.

Neutrophils and monocytes have a similar ability to phagocytose pathogens *in vivo* (33); here we initially examined if they also have similar downstream phagosomal environments. Neutrophils and monocyte purity following isolation was confirmed by both flow cytometry and morphological examination (Figures 1A,B). Sixty-eight percentage (SEM ± 4.3%) of neutrophils and 48% (±4.2%) of monocytes phagocytosed SNARF-labeled *Candida*. Neutrophil phagosomes were alkalinized to a pH of approximately 8.5 which was maintained for up to 30 min. Similarly, the phagosomes of monocytes also became alkaline, although to a lesser degree reaching and maintaining a pH of ~7.7 (Figure 1C). Monocytes also had slightly lower phagocytosis index than neutrophils but was not statistically significant (Figure 1D). CGD neutrophils displayed no differences in uptake in comparison to healthy controls, while the oxidase inhibitor, diphenyleneiodonium (DPI), decreased phagocytosis in neutrophils most likely due to off target effects.

Previous studies have demonstrated the inhibition of NOX2 activity caused phagosomal acidification (34, 35). To assess whether NOX2 regulates phagosomal pH, cells were treated as above in the presence DPI (36). As mentioned above DPI exhibits non-specific effects, while X-linked CGD patients have a specific defect in NOX2. Here, we used cells isolated from a CGD patient to confirm the inhibition of NOX2 with DPI. Oxidase activity, measured by Amplex red oxidation was slower in monocytes as previously described (37, 38) (Figures 1E,F). Taken together, these data highlight a similarity of monocyte and neutrophil phagosomes. However, the question arises, is there differences in the phagosome pH between circulating monocyte subsets?

**Figure 2:**
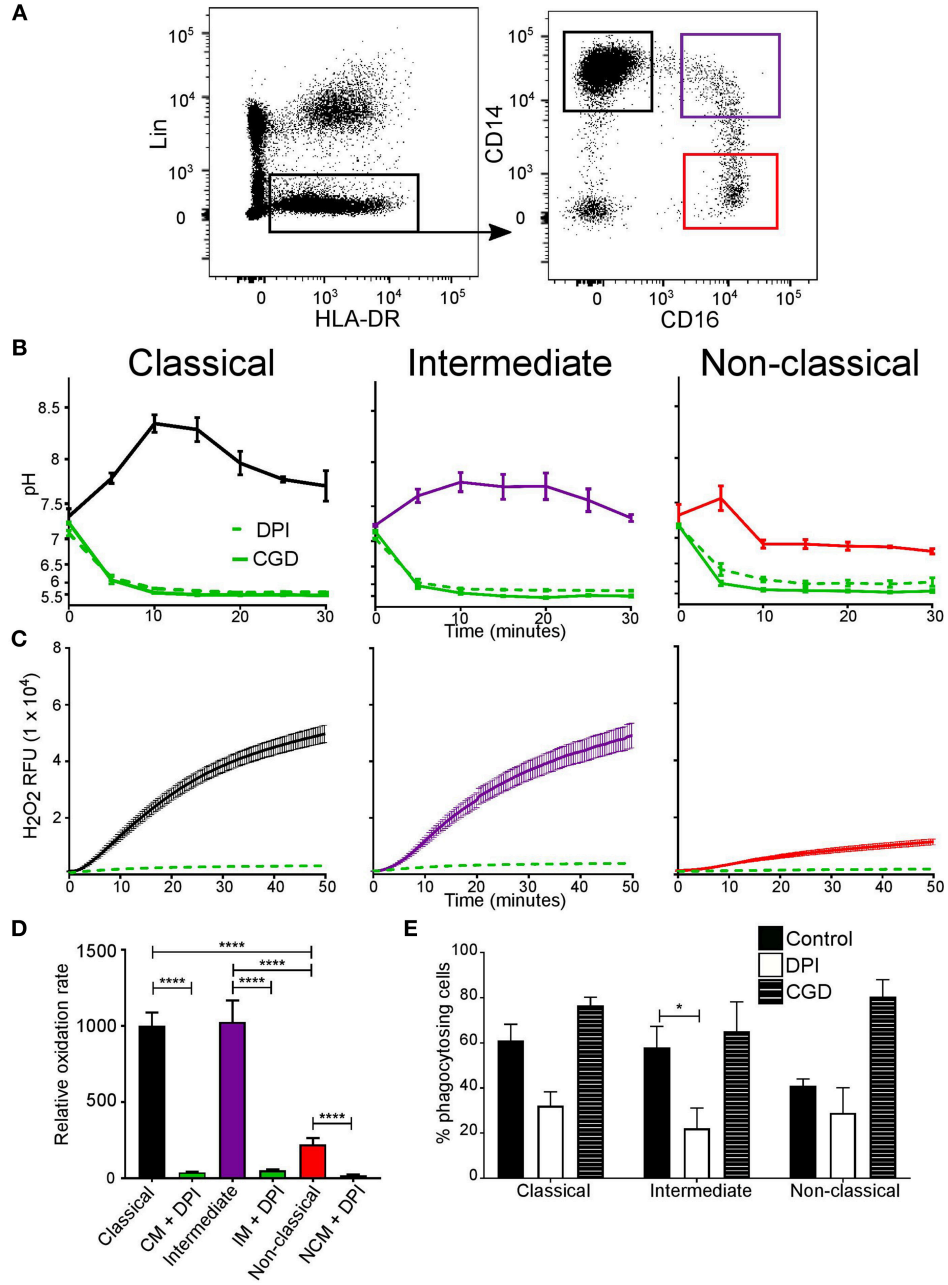
Phagosomal pH and NOX2 activity differed between monocyte subsets. **(A)** FACS isolation gating strategy of the three monocyte subsets based on CD14 and CD16 expression (shown in colored boxes). Lineage cells which expressed CD1c, CD3, CD11c, CD19, CD20, CD56, CD66b, or CD123 were excluded. **(B)** Time course of changes in phagosomal pH in monocyte subsets over the first 30 min after phagocytosis. The effects of 5 μM DPI (*n* = 3, dashed green line) and measurements from an X-linked CGD patient cells (*n* = 1, solid green line) are also shown. **(C)** Respiratory burst activity in subsets in response to stimulation with PMA, mean (solid line) ± SEM (dashed line). **(D)** Compiled results of the maximum rates from **(C)** mean ± SEM. **(E)** Quantitation of phagocytosis from experiments in **(B)**, mean ± SEM. Calculated *p*-values from one-way ANOVA with Bonferroni post-test analysis: ^*^p < 0.05, ^****^p < 0.0001. The statistics for phagosomal pH **(B)** between monocyte control conditions for each subset for pH was *p* < 0.001, and also for control vs. DPI and CGD. No statistical significance was found between DPI and CGD for all subsets. For the respiratory burst **(C)**, no significance was found between classical and intermediate controls, but *p* < 0.001 for classical and intermediate controls vs. DPI, and *p* < 0.01 for non-classical control vs. DPI.

To address this, monocyte subsets were sorted by FACS to explore if differences in phagosomal pH and respiration exist (Figure 2A). Interestingly, the phagosomal pH in all three subsets were distinct (Figure 2B). The phagosomes of classical monocytes alkalinised to around pH 8.5 by 10 min, after which the pH gradually fell to around 7.7. While, non-classical monocytes showed a brief alkalinization at about 5 min after which the pH fell to below 7.0 and remained slightly acidic. The pH of intermediate monocyte phagosomes was in-between that of the other two subsets.

As in neutrophils, the alkalinization of the phagosomes was produced through the action of NOX2 since the vacuolar pH of all monocyte subsets was acidic obtained from CGD cells or following treatment with DPI (Figure 2C). The rate of the respiratory burst was similar for the classical and intermediate monocytes while slower in non-classical monocytes. In all subsets, respiration was significantly inhibited by DPI (Figure 2D). On the other hand, phagocytic capacity remained equal (around 50%) (Figure 2E), DPI only affected phagocytosis in intermediate monocytes.

**Figure 3:**
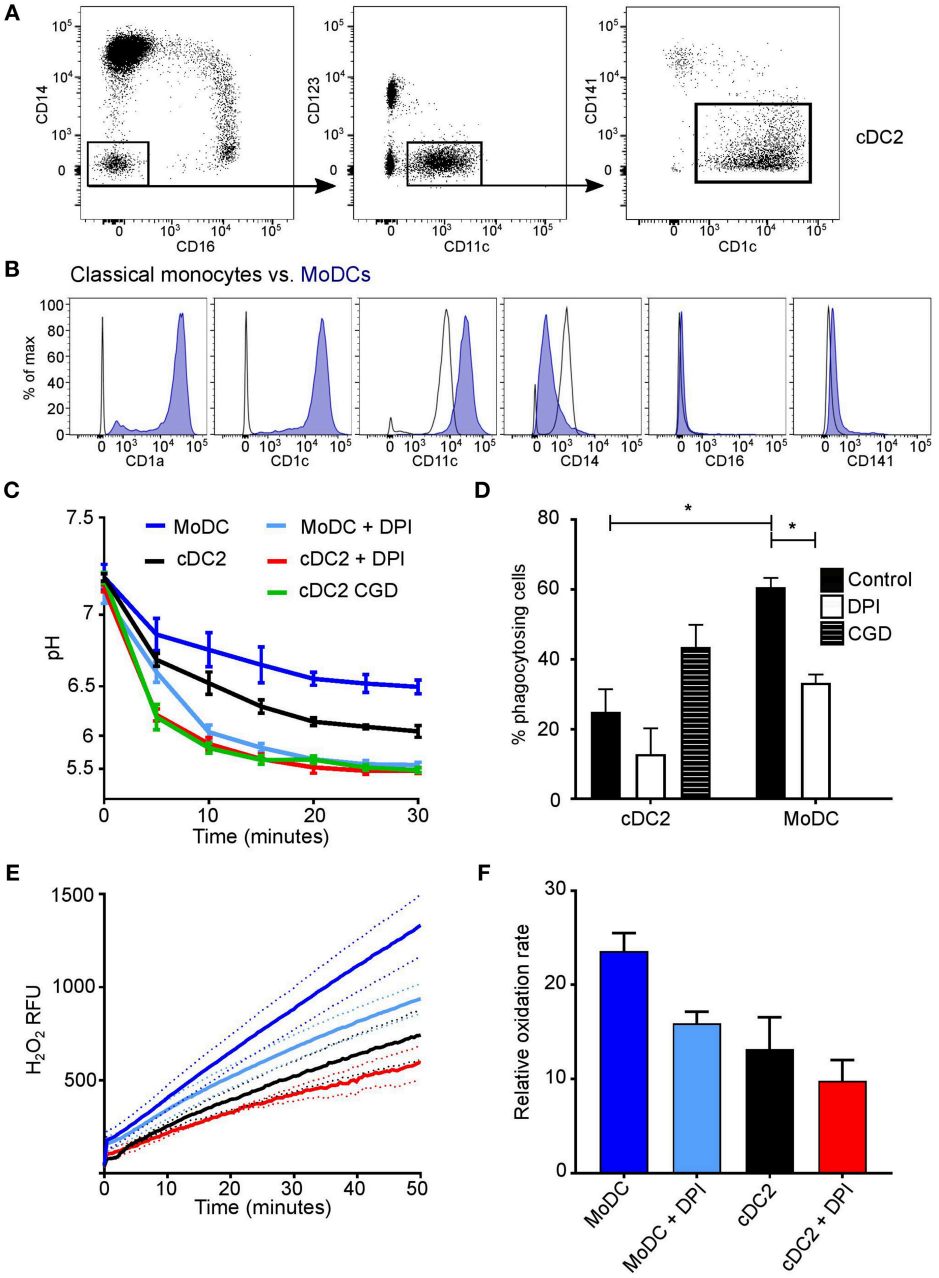
Comparison of cDC2 and monocyte-derived DCs. **(A)** Gating strategy used to isolate cDC2 (CD1c^+^ DCs) using FACS; **(B)** *in vitro* derived-MoDC were phenotypically validated according to surface markers by comparison with their precursor classical monocytes (40, 41), a representative plot is shown based on three experiments; **(C)** Time course of changes in the pH in the phagosomes of MoDC and cDC2 mean ± SEM, from a healthy subject, with and without DPI (*n* = 3), and cDC2 from a CGD patient (*n* = 1); **(D)** quantitation of phagocytosis from **(C)**, mean ± SEM. **(E)** NADPH oxidase activity in response to PMA stimulation in MoDs and cDC2 in the presence and absence of 5 μM DPI, mean (solid line) ± SEM (dashed line); **(F)** maximal rates of respiration from **(E)**, mean ± SEM. Calculated *p*-values from one-way ANOVA with Bonferroni post-test analysis: ^*^p < 0.05. For statistics of phagosomal pH **(C)** *p* < 0.05 for cDC2 vs. MoDC; *p* < 0.001 for cDC2 vs. DPI and for cDC2 vs. CGD; *p* < 0.001 for MoDC vs. DPI. For the respiratory burst **(E)**: *p* < 0.01 for cDC2 vs. MoDC; no significance was found between cDC2 and cells with DPI, but *p* < 0.001 for MoDC vs. DPI.

At least three subsets of DCs can found in human peripheral blood (39, 40), plasmacytoid DCs (pDC), cDC1 (CD141^+^), and cDC2 (CD1c^+^) (Figure 3A). Upon examination of DC phagosomes we were unable to detect phagocytosis of the SNARF-labeled *Candida* in the pDC fraction, and could not obtain sufficient cDC1 for their adequate examination. Therefore, the cDC2 population was examined and compared with *in vitro* monocyte-derived DC (Figure 3B), which are believed to mimic monocyte-derived cells that differentiate into dendritic-like cells when entering inflamed tissues. It was important to compare primary cDC with *in vitro* monocyte-derived DC as these cells have been an invaluable tool to many research groups in lieu of primary cDC (due to their relative scarcity). Yet, it is important to note, the ontogeny (42, 43) and transcriptome (44) are distinct between naturally occurring cDC and monocyte-derived DC.

We found that the phagosomal pH was slightly less acidic in MoDC compared with primary circulating cDC2 (Figure 3C). Upon the addition of DPI, the pH decreased further (*p* < 0.001 in both cDC2 and MODC types), resembling levels observed in cDC2 from the CGD patient. Interestingly, cDC2 were much less phagocytic than MoDC, with a mean percentage of 23.4 ± 4.6 SEM in comparison with 60.0 ± 3.1, respectively, which may correspond with previous findings that MoDC are superior at receptor mediated endocytosis of immune complexes (28).

The amount and rate of hydrogen peroxide production in response to PMA was lower in both cDC2 and MoDC (Figures 3E,F) in comparison to neutrophils and monocytes (Figures 1E,F). MoDC produced significantly more H_2_O_2_ than cDC2 (*p* < 0.01), which was further lowered by DPI (*p* < 0.001), however was not significant in cDC2.

**Figure 4:**
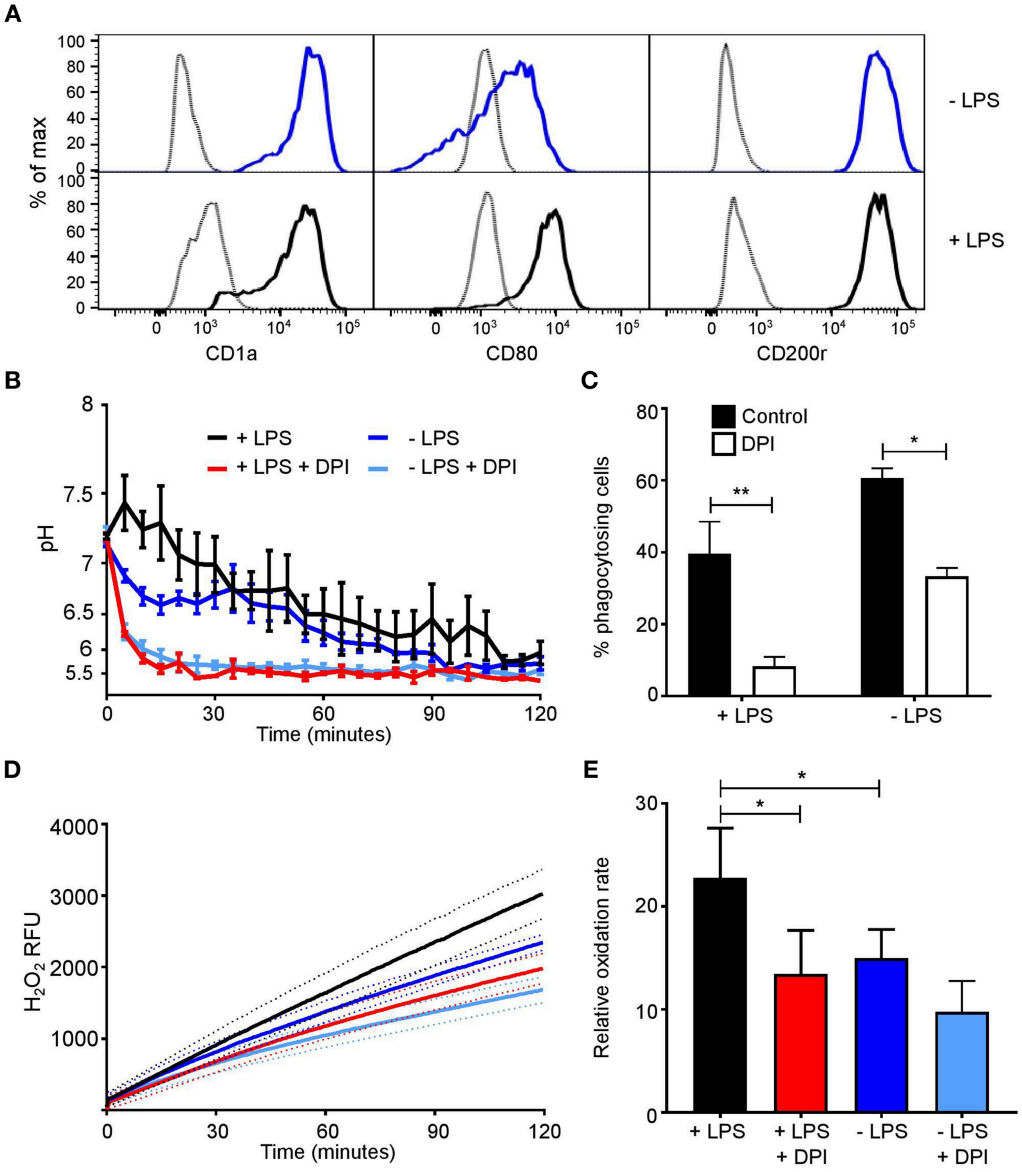
MoDC stimulated with LPS have more alkaline phagosomes and greater NADPH oxidase activity than untreated MoDC. **(A)** Flow cytometry phenotypic analysis of differentiation in untreated and LPS-treated MoDC. Gray, unstained controls; dark blue, untreated MoDC; black line, LPS + MoDC. **(B)** Time course of changes in phagosomal pH over 2 h in cells with and without 5 μM DPI, mean ± SEM. **(C)** Quantitation of phagocytosis from **(B)**, mean ± SEM. **(D)** Time course of changes in NADPH oxidase activity in response to PMA stimulation, mean (solid line) ± SEM (dashed line); **(E)** relative oxidation rate calculated from the final 30 min of **(D)**. Calculated *p*-values from one-way ANOVA with Bonferroni post-test analysis: ^**^p < 0.01, ^*^p < 0.05, *n* = 3. Statistics for **(B)** and **(D)** *p* < 0.0001 between control untreated and LPS-treated MoDC, and *p* < 0.0001 between control and DPI-treated cells.

Differences between our data and others who reported the phagosomal pH to be alkaline could be accounted for by LPS treatment. We were able to alter the activity of MoDC with 1 μg/ml LPS for 24 h (27, 29, 45). Phenotypic changes were first investigated looking at CD1a, CD80, and CD200r expression (Figure 4A). Both untreated and LPS-treated cells had a high expression of CD1a and CD200r, while LPS-treated MoDC had increased expression of CD80 in comparison to untreated cells. Measurements of the functional assays were increased to 2 h in line with other studies (7, 46, 47). Within the first 30 min, LPS-treated MoDC had a more alkaline phagosomal pH than untreated MoDC peaking at pH 7.45 at 5 min, after which both cell conditions further acidified to pH 5.5 (Figure 4B). DPI caused immediate acidification in both cell types. Fewer LPS-treated MoDC phagocytosed opsonized *Candida* than non-treated MoDC, but not by a significant margin. Interestingly, DPI adversely affected phagocytosis in both cell types (Figure 4C).

Initially, no marked difference was observed in respiratory burst between treated and untreated cells within the first 50 min (Figure 4D), at 2 h treated MoDC produced a higher H_2_O_2_-induced fluorescence than untreated MoDC (*p* < 0.0001). LPS-treated cells maintained a linear rate over the recorded 2 h, while in untreated cells the rate started to decline from 30 min onwards. The relative oxidation rate (Figure 4E) was calculated over the last 30 min of the time course to highlight this difference. The measured increase at this endpoint between naïve and LPS-treated MoDC was ~22%.

**Figure 5:**
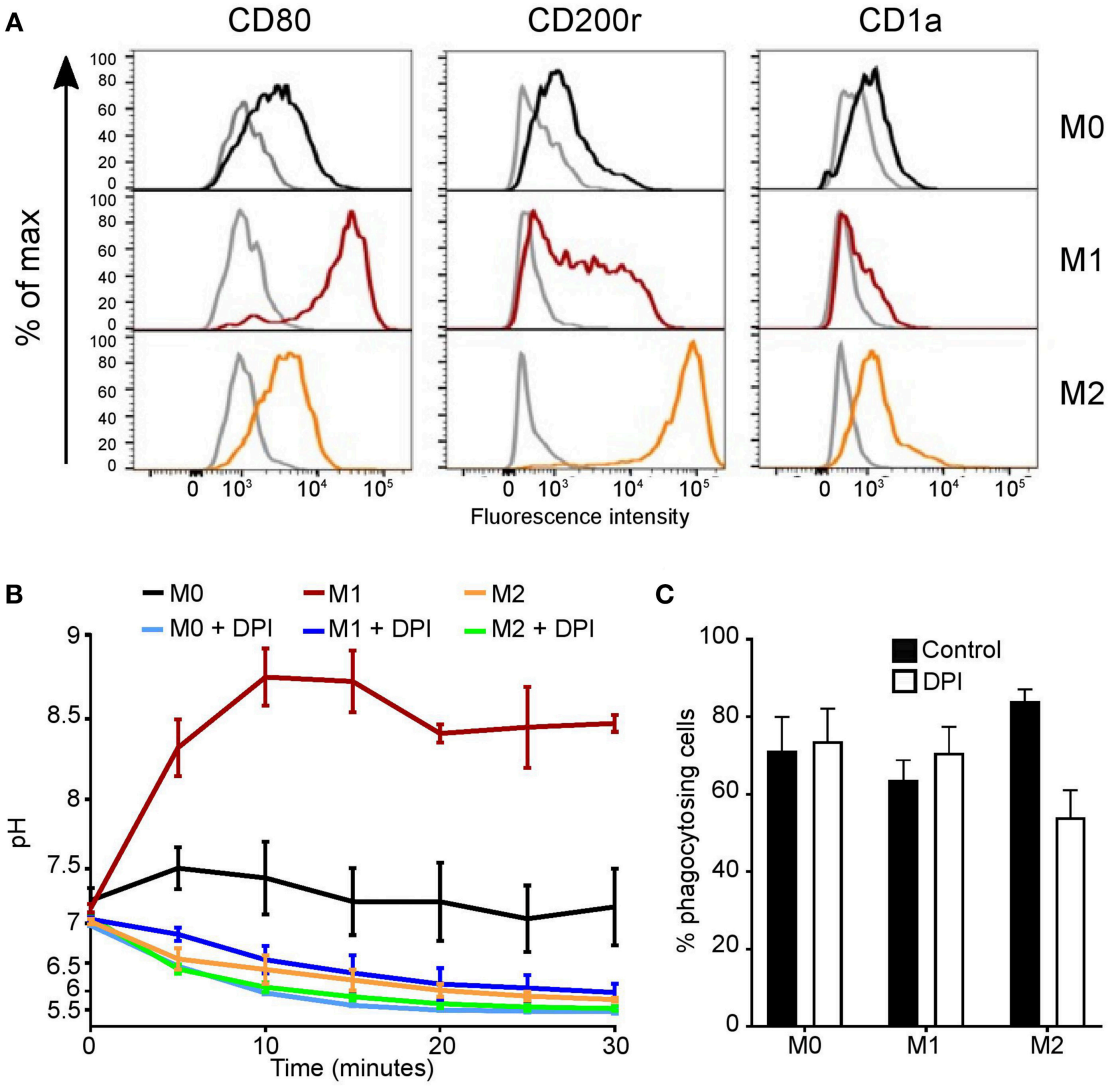
Polarized monocyte-derived cells have different phagosomal pH profiles. **(A)** Phenotypic validation by flow cytometry of monocyte-derived cells differentiation from undifferentiated “M0” cells into classically activated “M1” and alternatively activated “M2” monocyte-derived cells. CD80 was used as a marker for M1 differentiation, CD200r for M2, and CD1a to exclude dendritic cells differentiation. Isotype controls are shown in gray, representative of *n* = 3. **(B)** Time course of phagosomal pH in the three monocyte-derived cell populations with and without 5 μM DPI, mean ± SEM, *n* = 3. **(C)** Quantitation of phagocytosis from **(B)**, mean ± SEM, *n* = 3. Calculated *p*-values from one-way ANOVA with Bonferroni post-test analysis: for phagosomal pH **(B)** *p* < 0.001 for between all controls, *p* < 0.001 for M1 vs. M1 DPI and M0 vs. M0 DPI, ns for M2 vs. M2 DPI.

Finally, we measured the phagosomal pH of “M1/classically activated” and “M2/alternatively activated” monocyte-derived cells and undifferentiated state “M0” cells. We first confirmed that the identity of these *in-vitro* polarized cells (Figure 5A). The phagosomal pH of “M1” monocyte-derived cells, were alkaline at 5 min following phagocytosis, similar to neutrophils, and maintained a phagosomal pH of ~8.5 (Figure 5B). In contrast, “M2” monocyte-derived cells acidified their phagosomes in a similar fashion to non-classical monocytes. “M0” cells reached a pH between that of “M1” and “M2” monocyte-derived cells, this was due to both acidic and alkaline phagosomes. There was no significant change in pH between “M2” cells with and without DPI, suggesting this is independent of NOX2–these data corroborate previous elegant studies conducted by Canton et al. (6). There were no significant differences in phagocytosis index between these cells with and without DPI (Figure 5C).

## Discussion

The phagosome in professional phagocytes falls into two main categories, pathogen killing and digestion, or antigen processing, and presentation. Both these roles require the activity of digestive enzymes and these in turn are highly dependent upon the conditions within the phagocytic vacuole.

It is known that the neutrophil and monocyte cytoplasmic granules contain a variety of enzymes, including lysozyme, and myeloperoxidase (48–50), although more poorly classified in monocytes. The pH optima varies between such enzymes for example myeloperoxidase tested in neutrophils is pH 5–6 (1) whereas, lysozyme (tested in serum) lies between pH 8–9 (51). Our observation of differences in phagosomal pH between monocyte subsets, may indicate that (a) these enzymes are present in different subsets of monocytes, (b) they are contained within different granules which degranulate at different times in the evolution of the vacuole, or (c) that the timing of their activity varies with temporal changes in the vacuole.

Our results highlight that, like neutrophils, classical monocytes develop alkaline vacuoles. In addition, the duration and extent of vacuolar alkalinization varies considerably between monocyte subsets, suggesting functional diversity between the cells.

The results on polarized monocyte-derived cells mirrored those described by Grinstein (6), in addition we also observed non-polarized “M0” monocyte-derived cells were more acidic than the “M1” cells yet distinct from the “M2” monocyte-derived cells. The phagosomal pH of “M1” and “M0” cells were acidified significantly with the addition of DPI, whereas this was not true for “M2” monocyte-derived cells. It is apparent that the mechanism regulating phagosomal pH differs between monocyte-derived cells states, either dependent or independent of NOX2, consequently warranting further investigation.

The role of the NADPH oxidase and antigen handling by dendritic cells remains a point of contention. A number of groups report that mouse bone marrow-derived DC (46, 52) and human blood DC (53) have an alkaline phagosomal pH (pH 7–8), elevated by the NADPH oxidase, which is necessary for optimal antigen processing and presentation. On the other hand, Rybicka et al. (8) reported that mouse BMDC and splenic DCs had acidic phagosomal pHs that were unaltered by the activity of the oxidase, and proposed that it was the reducing environment, indirectly linked to the pH, rather than the proton concentration that was important for antigen presentation. Similarly, others have observed comparable results also using mouse BMDC (54). In human MoDC several groups have reported an acidic phagosomal pH following phagocytosis (47, 55).

In accordance with these findings, we did not observe the vacuolar pH of either naïve cDC2 or the majority of MoDC to become alkaline during our analysis. However, when we stimulated the cells with LPS, the phagosomes of many MoDC became alkaline. Additionally, the NADPH oxidase activity was higher in these cells than untreated MoDC. This observation is in line with studies by Savina et al. (56), who demonstrated NADPH oxidase influences the vacuolar pH, further supported in both MoDC and cDC2 when the oxidase was absent in CGD or inhibited by DPI, but further work is required to help understand the relationship between phagosomal pH and DC function.

There is also the possibility that different pathogens alter the phagosome, for instance, *Mycobacterium* infection in macrophages change the phagosomal pH by interfering with the onset of acidification (57, 58). In this study, we utilized dead yeast organisms as a tool to assess phagosomal pH, as live organisms do not efficiently retain SNARF-1. We hope that further technical advances will help elucidate the effect of live organisms on the phagosome.

Here, we demonstrate differences in the vacuole environments between neutrophils, monocytes, dendritic cells, and monocyte-derived cells. This will consequently have downstream effects on the enzymology within these compartments. To further explore tissue macrophage heterogeneity, primary macrophages from distinct tissue i.e., lung alveolar macrophages or skin Langerhans cells could be examined. Future work identifying enzymes released into the vacuoles, will enable a better understanding of the function of these cells.

## Author Contributions

JF and AP isolated and cultured cells and performed flow cytometry experiments. AP performed purity analyses. JF performed and analyzed microscopy experiments and Amplex UltraRed assays and made the figures. SY and AS designed the research, all authors wrote the paper.

### Conflict of Interest Statement

The authors declare that the research was conducted in the absence of any commercial or financial relationships that could be construed as a potential conflict of interest.
